# Fast Fusion Clustering via Double Random Projection

**DOI:** 10.3390/e26050376

**Published:** 2024-04-28

**Authors:** Hongni Wang, Na Li, Yanqiu Zhou, Jingxin Yan, Bei Jiang, Linglong Kong, Xiaodong Yan

**Affiliations:** 1 School of Statistics and Mathematics, Shandong University of Finance and Economics, Jinan 250014, China; wanghongni@mail.sdufe.edu.cn (H.W.); lina@sdufe.edu.cn (N.L.); 2 School of Science, Guangxi University of Science and Technology, Liuzhou 545006, China; 100002146@gxust.edu.cn; 3 Academy of Mathematics and Systems Science, Chinese Academy of Sciences, Beijing 100190, China; yanjingxin22@mails.ucas.ac.cn; 4 Department of Mathematical and Statistical Sciences, University of Alberta, Edmonton, AB T6G 2G1, Canada; bei1@ualberta.ca; 5 Zhongtai Securities Institute for Financial Studies, Shandong University, Jinan 250100, China

**Keywords:** unsupervised learning, random projection, ADMM algorithm, fusion clustering

## Abstract

In unsupervised learning, clustering is a common starting point for data processing. The convex or concave fusion clustering method is a novel approach that is more stable and accurate than traditional methods such as *k*-means and hierarchical clustering. However, the optimization algorithm used with this method can be slowed down significantly by the complexity of the fusion penalty, which increases the computational burden. This paper introduces a random projection ADMM algorithm based on the Bernoulli distribution and develops a double random projection ADMM method for high-dimensional fusion clustering. These new approaches significantly outperform the classical ADMM algorithm due to their ability to significantly increase computational speed by reducing complexity and improving clustering accuracy by using multiple random projections under a new evaluation criterion. We also demonstrate the convergence of our new algorithm and test its performance on both simulated and real data examples.

## 1. Introduction

Clustering is a pivotal technique in unsupervised learning, applied extensively across various scientific and technological fields that handle large datasets. Clustering also plays a crucial role in data labelling, which sets the stage for the application of artificial intelligence and machine learning models [1,2] on the organized data to perform predictive analytics and classification tasks. Traditional clustering algorithms like k-means, Gaussian mixture models, and hierarchical clustering often face stability challenges due to their concave optimization formulations, which can lead to variability in results due to factors such as initial conditions or data outliers [3,4,5]. Recent advancements in convex or concave fusion methods have shown promise in enhancing stability, achieving more consistent global or local optimality and reliable estimation of cluster centers and counts through sparse-inducing penalties on pairwise centers [6,7,8,9]. For clustering high-dimensional data, the data can be mapped into a high-dimensional feature space (kernel space) for processing [10], or clustering can be achieved by optimizing a smooth and continuous objective function that is based on robust statistics [11]. This paper introduces a comprehensive empirical validation of these methods across simulation studies and real data analysis, detailing their improved stability over traditional methods and the practical implications of these advancements.

In fusion clustering, *p*-dimensional observations $X_{i}$, i=1,…,n are each parameterized by their own centroid $\mu_{i}$. These centroids are estimated under the assumption that all observations can be grouped into *K* clusters $G_{1},\ldots ,G_{K}$, such that for $i\in G_{k}$, $\mu_{i}=\rho_{k}$, where $\rho_{k}$ represents the cluster center for observations in cluster $G_{k}$. Fusion clustering aims to concurrently estimate the cluster centroids $\rho_{k}$ and the partitions $G_{k}$ by minimizing the following objectives

(1) $$\frac{1}{2}\sum_{i=1}^{n}∥X_{i}-\mu_{i}{∥}^{2}+\sum_{i<j}p_{\lambda }(∥\mu_{i}-\mu_{j}{∥}_{\tau }).$$

The penalty function $p_{\lambda }(∥\cdot ∥\tau )$ is used to control the complexity of the model, and it is determined by the tuning parameter λ. The form of the norm used is represented by ∥·∥τ. This penalty function is typically used in fusion clustering to encourage sparsity in the estimated cluster centroids.

The penalty function $p_{\lambda }(∥\cdot ∥\tau )$ controls the complexity of the model and is determined by the tuning parameter λ. The norm used is ∥·∥τ. The penalty function is typically used in fusion clustering to promote sparsity in cluster centroids.

Convex fusion clustering methods have been widely studied due to their computational simplicity and ability to find global optima. These methods often employ $\ell_{1}$, $\ell_{2}$, or $\ell_{\infty }$ penalties as the penalty function $p_{\lambda }{(∥\cdot ∥}_{\tau })$ [12,13,14,15,16,17]. However, convex fusion can lead to biased estimates of the individual centroids, resulting in solutions with a large number of dense clusters [18,19]. To address this issue, researchers have proposed using concave fusion clustering methods, such as those using minimax concave penalties (MCPs) [20], truncated Lasso penalties (TLPs) [8], and arbitrary concave penalties.

While robust, convex and concave fusion clustering methods are computationally demanding with a $O(n^{2}p)$ complexity, which can limit their practicality in scenarios involving large sample sizes *n* and high-dimensional datasets *p*. This article proposes a strategy for overcoming this limitation using random projection techniques [21,22,23,24]. The approach involves the construction of a random diagonal matrix whose diagonal elements are sourced from a binary distribution. This matrix is then projected onto the pairwise component of the fusion method. By doing so, the number of pairwise differences between individual centroids, $∥\mu_{i}-\mu_{j}∥$, is substantially reduced. This reduction not only decreases the computational load but also maintains the integrity of the clustering process, enhancing the algorithm’s scalability without excessively increasing the operational overhead. We provide empirical evidence demonstrating that this method significantly reduces the computational time while preserving the clustering quality, as shown in our simulation section.

In unsupervised learning, rapid clustering processes are crucial for handling large datasets efficiently. Our study introduces a novel approach to fusion clustering to enhance computational speed without compromising accuracy. Our contributions are summarized as follows: (1) We propose using random projection techniques to simplify the fusion aspect of clustering, effectively diminishing the pairwise centroids discrepancies and significantly boosting computational efficiency by minimizing the fusion step’s complexity. (2) We have developed a novel double recursive random projection ADMM method designed for efficient high-dimensional fusion clustering, improving the accuracy of clustering.

In the remainder of this paper, the proposed new ADMM algorithm will be described in Section 2. This section will also include an analysis of the computational complexity and convergence of the algorithm. It will also include a strategy for improving cluster accuracy. The finite-sample properties of the proposed new ADMM algorithm will be evaluated through simulation studies in Section 3, and the method will be demonstrated using a real data example in Section 4. Concluding remarks will be presented in Section 5, and technical proofs will be provided in the Appendix A and Appendix B.

## 2. Methodology

To improve convex or concave fusion clustering efficiency, we propose an extension of the classical ADMM algorithm based on a random projection called RP-ADMM. A random projection can significantly reduce the time and computational resources needed to analyze high-dimensional data, making it suitable for large datasets and real-time processing. In this section, we will discuss the RP-ADMM algorithm’s computational complexity and convergence.

### 2.1. Random Projection Based ADMM

Previous ADMM algorithms for convex or concave fusion clustering [6,8] have suffered from a high computational burden due to the need to consider all n(n−1)/2 pairwise differences between individual centroids. This is represented by the fusion matrix $E={\left\{\left(e_{i}-e_{j}\right),i<j\right\}}_{\frac{n(n-1)}{2}\times n}^{T}$, where $e_{i}$ is the *i*th unit vector with a 1 in the *i*th position and 0s elsewhere, and $e_{i}-e_{j}$ can be interpreted as the difference between the *i*th and *j*th individual centroids. The computational complexity of this approach is $O(n^{2})$, which becomes infeasible for large sample sizes *n*.

**Bernoulli distribution-based random projections ADMM**  

It is worth noting that pairwise differences between individual centroids can be deduced from other differences. For example, if we know that $\mu_{1}-\mu_{2}=0$ and $\mu_{2}-\mu_{3}=0$, we can conclude that $\mu_{1}-\mu_{3}=0$. This means that it may be unnecessary to consider the row $e_{1}-e_{3}$ in E. To reduce the computational burden of convex or concave fusion clustering, we propose a random projection approach. This only considers a small subset of the n(n−1)/2 pairwise differences between individual centroids. This is achieved by generating indicators $\pi_{ij}$ from a Bernoulli distribution with probability α. We then form a random matrix Π, which is a diagonal matrix with diagonal elements ${\left(\pi_{12},\cdots ,\pi_{1n},\pi_{23},\cdots ,\pi_{2n},\cdots ,\pi_{(n-1)n}\right)}^{T}$. If $\pi_{ij}=1$, the difference between $\mu_{i}$ and $\mu_{j}$ is taken into account; if $\pi_{ij}=0$, it is not considered. The probability α controls the size of the subset of pairwise differences considered. The matrix ΠE can be seen as a projection of E onto a sparse matrix. This is with about n(n−1)(1−α)/2 rows being zero vectors and about n(n−1)α/2 ones being nonzero vectors. This projection is based on a Bernoulli distribution. Finally, we form a new fusion matrix Ω by deleting the rows of zero vectors in ΠE. The new fusion matrix is given by $\Omega ={\left(\Omega_{1},\cdots ,\Omega_{\kappa }\right)}^{T}$, where $\Omega_{j},$j=1,⋯,κ, denotes *j*th row vector of Ω.

 We just consider τ=2 in (1) for simplicity and propose a random projection-based fusion criterion by

(2) $$\ell_{p}\left(\mu ;\lambda \right)=\frac{1}{2}\sum_{i=1}^{n}∥X_{i}-\mu_{i}{∥}^{2}+\sum_{i<j}\pi_{ij}p_{\lambda }(∥\mu_{i}-\mu_{j}∥),$$

where $\mu ={\left(\mu_{1},\cdots ,\mu_{n}\right)}_{n\times p}^{T}$. Furthermore, the objective function in (2) is equivalent to

(3) $$\begin{array}{cc} {\tilde{\ell }}_{p}\left(\mu ,\phi ;\lambda \right) & =\frac{1}{2}{∥X-\mu ∥}_{F}^{2}+\sum_{j=1}^{\kappa }p_{\lambda }\left(|\right|\phi_{j}||),\;\\ \;\mathrm{subject}\;\mathrm{to}\; & \Omega \mu -\phi =0, \end{array}$$

where $X={\left(X_{1},\cdots ,X_{n}\right)}^{T}$, $\phi ={\left(\phi_{1},\cdots ,\phi_{\kappa }\right)}_{\kappa \times p}^{T}$. Under the constraints in (3), the augmented Lagrangian Q(μ,ϕ,η;λ) has the form

(4) $$\begin{array}{c} {\tilde{\ell }}_{p}\left(\mu ,\phi ;\lambda \right)+\sum_{j=1}^{\kappa }\eta_{j}^{T}\left(\mu^{T}\Omega_{j}-\phi_{j}\right)+\frac{\varphi }{2}{∥\Omega \mu -\phi ∥}_{F}^{2}, \end{array}$$

where the dual variables $\eta ={\left(\eta_{1},\cdots ,\eta_{\kappa }\right)}_{\kappa \times p}^{T}$ are Lagrange multipliers, and φ is a tuning parameter. Under the iterative value $\mu^{\left(m\right)}$ and $\eta^{\left(m\right)}$ at the *m*th step, we conduct the Bernoulli distribution-based random projection ADMM (RP-ADMM) iterative algorithm and compute the estimates of (ϕ,η,μ) as follows:

(5) $$\begin{array}{ccc} \phi^{\left(m+1\right)} & = & \arg\min_{\phi }L\left(\phi ,\mu^{\left(m\right)},\eta^{\left(m\right)};\lambda \right), \end{array}$$

(6) $$\begin{array}{ccc} \eta^{\left(m+1\right)} & = & \eta^{\left(m\right)}+\varphi \left(\Omega \mu^{\left(m\right)}-\phi^{\left(m+1\right)}\right), \end{array}$$

(7) $$\begin{array}{ccc} \mu^{\left(m+1\right)} & = & \arg\min_{\mu }Q\left(\mu ,\phi^{\left(m+1\right)},\eta^{\left(m+1\right)};\lambda \right), \end{array}$$

where $L(\phi ,\mu^{\left(m\right)},\eta^{\left(m\right)};\lambda )$ equals

(8) $$\frac{\varphi }{2}{∥\Omega \mu^{\left(m\right)}-\phi +\varphi^{-1}\eta^{\left(m\right)}∥}_{F}^{2}+\sum_{j=1}^{\kappa }p_{\lambda }\left(∥\phi_{j}∥\right),$$

and $Q(\mu ,\phi^{\left(m+1\right)},\eta^{\left(m+1\right)};\lambda )$ equals

(9) $$\begin{array}{cc} \; & {\tilde{\ell }}_{p}\left(\mu ,\phi^{\left(m+1\right)}\right)+\frac{\varphi }{2}{∥\Omega \mu -\phi^{\left(m+1\right)}∥}_{F}^{2}\\ \; & +\sum_{j=1}^{\kappa }\eta_{j}^{T(m+1)}\left(\mu^{T}\Omega_{j}-\phi_{j}^{\left(m+1\right)}\right). \end{array}$$

Ma and Huang (2017) [18] have argued that under (8), the element $\phi_{j}^{\left(m+1\right)}$ of $\phi^{\left(m+1\right)}$ is the minimizer of $\frac{\varphi }{2}\left|\right|\zeta_{j}^{\left(m\right)}-\phi_{j}{\left|\right|}^{2}+p_{\lambda }\left(|\right|\phi_{j}\left||\right)$, where $\zeta_{j}^{\left(m\right)}=\Omega_{j}^{T}\mu^{\left(m\right)}+\varphi^{-1}\eta_{j}^{\left(m\right)}$. For different thresholding operator $p_{\lambda }\left(\cdot \right)$, the estimate $\phi_{j}^{\left(m+1\right)}$ has different results. Such as,

- For the Lasso penalty [25],
  $$\begin{array}{c} \phi_{j}^{\left(m+1\right)}=S\left(\zeta_{j}^{\left(m\right)},\lambda /\varphi \right);\\ S\left(w,t\right)=\left\{\begin{array}{cc} (1-t/||w||)w, & \mathrm{if}\;t/||w||<1;\\ 0, & \mathrm{otherwise}. \end{array}\right. \end{array}$$
- For SCAD penalty [26] with a>1/φ+1,
  $$\begin{array}{c} \phi_{j}^{\left(m+1\right)}=\left\{\begin{array}{cc} S(\zeta_{j}^{\left(m\right)},\lambda /\varphi ), & \mathrm{if}\;||\zeta_{j}^{\left(m\right)}\left|\right|\leq \lambda +\lambda /\varphi ;\\ \zeta_{j}^{\left(m\right)}, & \mathrm{if}\;||\zeta_{j}^{\left(m\right)}\left|\right|>a\lambda ;\\ \frac{S(\zeta_{j}^{\left(m\right)},a\lambda /\left(\left(a-1\right)\varphi \right))}{1-1/((a-1)\varphi )}, & \mathrm{otherwise}. \end{array}\right. \end{array}$$
- For the MCP [27] with a>1/φ,
  $$\begin{array}{c} \phi_{j}^{\left(m+1\right)}=\left\{\begin{array}{cc} \frac{S(\zeta_{j}^{\left(m\right)},\lambda /\varphi )}{1-1/(a\varphi )}, & \mathrm{if}\;||\zeta_{j}^{\left(m\right)}\left|\right|\leq a\lambda ;\\ \zeta_{j}^{\left(m\right)}, & \mathrm{otherwise}. \end{array}\right. \end{array}$$
- For the TLP [8] with a>1,
  $$\begin{array}{c} \phi_{j}^{\left(m+1\right)}=\left\{\begin{array}{cc} S(\zeta_{j}^{\left(m\right)},\lambda /\varphi ), & \mathrm{if}\;||\zeta_{j}^{\left(m\right)}\left|\right|\leq a\lambda ;\\ \zeta_{j}^{\left(m\right)}, & \mathrm{otherwise}. \end{array}\right. \end{array}$$

Through some algebra, the problem of (9) is equivalent to the minimization of the function $h(\mu ,\phi^{\left(m+1\right)},\eta^{\left(m+1\right)})$, which has the from

$$\begin{array}{c} \frac{1}{2}{∥X-\mu ∥}_{F}^{2}+\frac{\varphi }{2}||\Omega \mu -\phi^{\left(m+1\right)}+\varphi^{-1}\eta^{\left(m+1\right)}{\left|\right|}_{F}^{2}. \end{array}$$

Under the given value of $\phi^{\left(m+1\right)}$, $\eta^{\left(m+1\right)}$, the updated $\mu^{\left(m+1\right)}$ are

$$\begin{array}{ccc} \mu^{\left(m+1\right)} & = & {\left(\varphi \Omega^{T}\Omega +I_{n}\right)}^{-1}\left(X+\varphi \Omega^{T}\left(\phi^{\left(m+1\right)}-\varphi^{-1}\eta^{\left(m+1\right)}\right)\right) \end{array}$$

where $I_{n}$ is n×n identity matrix. $\mu^{\left(m+1\right)}$ and $\phi^{\left(m+1\right)}$ are updated according to the random projection ADMM iterative algorithm (5)–(7) until the input of some convergence criteria, such as both dual and primal residuals being close to zero [28] in our practice. The convergence time of ADMM is highly related to the penalty parameter φ. A poor selection of φ can result in a slow convergence for the ADMM algorithm [29] and thus RP-ADMM. In this paper, we fix φ=1 throughout for simplicity.

To facilitate the updates of $\left(\phi^{\left(m+1\right)},\eta^{\left(m+1\right)},\mu^{\left(m+1\right)}\right)$ at the (m+1)th step in (5) to (7) of the RP-ADMM iterative algorithm, we need to specify a proper initial value (warm start). Here, we set $\eta^{\left(0\right)}=0$, $\phi^{\left(0\right)}=\Omega \mu^{\left(0\right)}$ and obtain the initial estimators $\mu^{\left(0\right)}={\left(\lambda^{★}\Omega^{T}\Omega +I_{n}\right)}^{-1}X$ as the minimizer of a ridge fusion criterion

(10) $$\begin{array}{c} \frac{1}{2}{∥X-\mu ∥}_{F}^{2}+\frac{\lambda^{★}}{2}{∥\Omega \mu ∥}^{2}. \end{array}$$

We summarize the above analysis in Algorithm 1.
**Algorithm 1** RP-ADMM for fusion clustering**Input**: data $X_{1},\cdots ,X_{n}$; Initialize $\mu^{\left(0\right)}$, $\eta^{\left(0\right)}$; tuning parameter, λ**Output**: an estimate of μ **for** m=0,1,2,⋯ **do**  compute $\phi^{\left(m+1\right)}$ using (5)  compute $\eta^{\left(m+1\right)}$ using (6)  compute $\mu^{\left(m+1\right)}$ using (7)  **if** convergence criterion is met, **then**   Stop and denote the last iteration by $\hat{\mu }\left(\lambda \right)$,  **else**   m=m+1  **end if**  **end for**

Practically, we would not want to conduct the RP-ADMM updates comprehensively until convergence to save computing time in the first iterations. Another trick is to adopt the initial values of subsequent convex relaxations as optimal values from the previous relaxed convex problem, which significantly reduces the number of RP-ADMM iterations.

### 2.2. Selection of Optimal Tuning Parameter

For a given λ, the converging value $\hat{\mu }\left(\lambda \right)$ of the above RP-ADMM procedure is defined as

(11) $$\hat{\mu }\left(\lambda \right)={\mathrm{argmin}}_{\mu }\ell_{p}\left(\mu ;\lambda \right),$$

where $\ell_{p}\left(\mu ;\lambda \right)$ is defined in (2) and the optimal value of λ can be selected via a properly constructed data-driven criterion. In particular, we partition the support of λ into a grid of $\lambda_{\min}=\lambda_{0}<\lambda_{1}<\cdots <\lambda_{J}=\lambda_{\max}$, and for each $\lambda_{j}$, we compute a solution path of $\hat{\mu }\left(\lambda_{j}\right)$ and obtain $\hat{K}\left(\lambda_{j}\right)$ distinct cluster centroids $\left\{{\hat{\rho }}_{1}\left(\lambda_{j}\right),\ldots ,{\hat{\rho }}_{\hat{K}\left(\lambda_{j}\right)}\left(\lambda_{j}\right)\right\}$, The optimal $\hat{\lambda }$ is selected by minimizing a data-driven BIC, i.e., $\hat{\lambda }={\mathrm{argmin}}_{\lambda_{j};j=1,\ldots ,J}\mathrm{BIC}\left(\lambda_{j}\right)$, where

(12) $$\begin{array}{cc} \mathrm{BIC}(\lambda ) & =\log\left\{\frac{1}{np}{∥X-\hat{\mu }\left(\lambda \right)∥}_{F}^{2}\right\}\\ \; & +\left(\log\left(np\right)+2\log\left(p\right)\right)\hat{K}\left(\lambda \right)/n. \end{array}$$

Subsequently, we obtain the estimator $\hat{\mu }=\hat{\mu }\left(\hat{\lambda }\right)$, and the individuals can be separated into $\hat{K}=\hat{K}\left(\hat{\lambda }\right)$ clusters accordingly, i.e., ${\hat{G}}_{k}=\left\{i:{\hat{\mu }}_{i}={\hat{\rho }}_{k},i=1,\ldots n\right\}$, $k=1,\ldots ,\hat{K}$.

**Figure 1:**
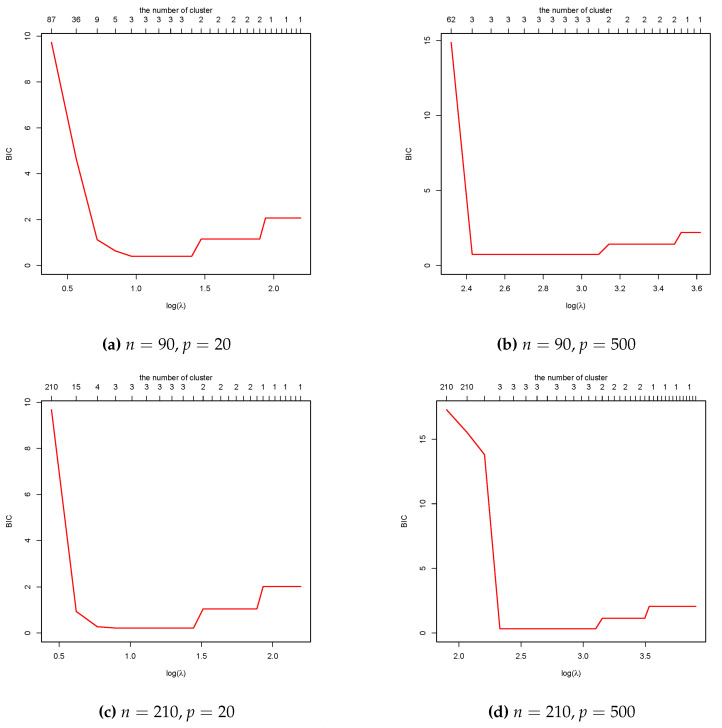
Plots of BIC values against log(λ) and the estimated cluster number of simulation with different *n*, *p* and true cluster number K=3.

Other methods for tuning parameters in clustering, such as generalized degrees of freedom with generalized cross-validation [8] and stability-based cross validation [25,30] can provide good results but may require extensive computation or the specification of a hyperparameter perturbation size [8]. In contrast, the proposed BIC is easy to compute and performs well in estimating cluster centroids and the true number of clusters (*K*). Figure 1 shows the change in BIC values against log(λ) and the cluster number of the simulation. Across all cases with different values of *n* and *p*, we observe that BIC(λ) decreases as the value of log(λ) increases. With recovering the true cluster number K=3, BIC($\hat{\lambda }$) reaches a minimum at the optimal $\hat{\lambda }$. Moreover, when log(λ) keeps increasing, the cluster centroids are continuously integrated, and BIC(λ) is enlarged. However, further research is needed to fully prove the consistency of the BIC in combination with the objective function (2).

### 2.3. Recursive RP-ADMM and Cluster Matrix

In the above cluster analysis, the effect of randomness on the clustering results was not considered. However, empirical analysis has shown that the impact of this randomness on the estimated cluster centers and numbers is minimal (i.e., ${\hat{\rho }}_{k}$’s and $\hat{K}$’s). However, the impact on the final partitioning results (i.e., which observations are grouped into a single cluster) can be significant. In response to this, we propose the Recursive RP-ADMM (RRP-ADMM) procedure, which performs multiple RP-ADMM cluster analyses by generating *M* random matrices (i.e., $\Omega_{m}$’s, m=1,⋯,M) and repeatedly conducting the analysis.

Once the multiple RP-ADMM cluster analyses have been completed, we must summarize the results. We define a n×n symmetric cluster matrix C where $C_{ij}=1$ denotes that the *i*th and *j*th observations belong to the same cluster; otherwise, $C_{ij}=0$. Another n×n symmetric matrix $\hat{D}$ is introduced, with element ${\hat{D}}_{ij}$ representing the relative frequency of the *i*th and *j*th observations belonging to the same cluster over the *M* independent RP-ADMM clustering procedures. The decision of whether the *i*th and *j*th observations should be grouped into a single cluster or not can then be treated as a classification problem, with the two possible class labels being 1 (belong to the same cluster) or 0 (do not belong to the same cluster). We can use an indicator function to transform the relative frequency into class labels and generate an estimator for the cluster matrix $\hat{C}$, i.e.,

(13) $$\begin{array}{c} \hat{C}=\left\{{\hat{C}}_{ij}:{\hat{C}}_{ij}=1_{\left({\hat{D}}_{ij}\geq 0.5\right)}\right\}, \end{array}$$

where $1_{\left(\cdot \right)}$ denotes the indicator function. We summarize the above procedure in Algorithm 2. This transformation can be understood as a voting-based aggregation strategy, similar to the one proposed by [31], which aims to reduce misclassification errors and improve the accuracy of the clustering. To evaluate the accuracy of the clustering results, we define a new measure called the similarity index (SI) between two data clusterings:

(14) $$\begin{array}{c} \mathrm{SI}=\frac{1}{n^{2}-n}∥\hat{C}{-C∥}_{1}=\frac{1}{n^{2}-n}\sum_{i=1}^{n}\sum_{j=1}^{n}\left|{\hat{C}}_{ij}-C_{ij}\right|. \end{array}$$

Like the Rand Index (RI) measure [32], the newly introduced evaluation criterion can be seen as a measure of the percentage of correct decisions made by some algorithm. The SI values also range from 0 to 1, with lower values indicating better algorithm performance.
**Algorithm 2** RRP-ADMM for fusion clustering**Input**: data $X_{1},\cdots ,X_{n}$; *M*; Initialize $\mu^{\left(0\right)}$, $\eta^{\left(0\right)}$; tuning parameter, λ**Output**: an estimate of μ  **for**m=0,1,⋯, *M* **do**  compute ${\hat{\mu }}^{\left(m\right)}$ using RP-ADMM **end for** **while** 1≤i≤n **do**  compute ${\hat{D}}_{ij}$ and ${\hat{C}}_{ij}$ from (13) **end while**

The classical convex or concave fusion clustering procedure in (1) requires $O(n^{2}p)$ operations and $O(n^{2}p+np)$ of storage for a single round of ADMM updates with primal and dual residual calculations, because all pairs of centroids are shrunk together in this method.

The RP-ADMM algorithm significantly improves computational efficiency compared to classical ADMM algorithm. It requires only O(κp+np) of storage, compared to $O(n^{2}p+np)$ for the classical ADMM algorithm, because the variables η and ϕ have only κ columns rather than n(n−1)/2. Additionally, the RP-ADMM algorithm requires only O(κp) operations for its most computationally demanding step, in comparison to $O(n^{2}p)$ for the classical ADMM algorithm. The RP-ADMM algorithm also requires O(κn) operations to conduct Cholesky factorization in every iteration, in comparison to $O(n^{3})$ for the classical ADMM algorithm. This efficient Cholesky factorization is computed only once and reused across repeated RP-ADMM updates.

At the end of this subsection, we will demonstrate the convergence of the RP-ADMM algorithm by showing that the sequence generated by the algorithm contains a subsequence that converges to a stationary point.

**Lemma** **1.** *Let ${\left\{\mu^{\left(m\right)},\phi^{\left(m\right)},\eta^{\left(m\right)}\right\}}_{k=1}^{\infty }$ be the sequence generated by Algorithm 1, then for some constant c>0,*

(15) $$\begin{array}{cc} \; & Q\left(\mu^{\left(m+1\right)},\phi^{\left(m+1\right)},\eta^{\left(m+1\right)}\right)-Q\left(\mu^{\left(m\right)},\phi^{\left(m\right)},\eta^{\left(m\right)}\right)\\ & \leq -\frac{c}{2}∥\mu^{\left(m+1\right)}-\mu^{\left(m\right)}{∥}^{2}+\psi {∥\eta^{\left(m+1\right)}-\eta^{\left(m\right)}∥}^{2} \end{array}$$

In order to prove that the sequence ${\left\{\mu^{\left(m\right)},\phi^{\left(m\right)},\eta^{\left(m\right)}\right\}}_{k=1}^{\infty }$ is convergent, we need to assume that $\phi^{\left(m\right)}$ is bounded and $\psi ∥\eta^{\left(m+1\right)-\eta^{\left(m\right)}}∥\to 0$ which are often observed in numerical tests.

**Theorem** **1.** *If ${\left\{\phi^{\left(m\right)}\right\}}_{k=1}^{\infty }$ are bounded and $\psi_{2}∥\nu^{\left(m+1\right)}-\nu^{\left(m\right)}{∥}_{F}+\psi_{1}{∥\eta^{\left(m+1\right)}-\eta^{\left(m\right)}∥}_{F}\to 0$, then ${\left\{\mu^{\left(m\right)},\phi^{\left(m\right)},\eta^{\left(m\right)}\right\}}_{k=1}^{\infty }$ is bounded. Moreover, there exist a subsequence ${\left\{\mu^{\left(k_{j}\right)},\phi^{\left(k_{j}\right)},\eta^{\left(k_{j}\right)}\right\}}_{k_{j}=1}^{\infty }$, such that*

$$\begin{array}{cc} \lim_{k_{j}\to \infty }(∥\mu^{\left(k_{j}+1\right)}-\mu^{\left(k_{j}\right)}∥ & +∥\phi^{\left(k_{j}+1\right)}-\phi^{\left(k_{j}\right)}∥\\ & +∥\eta^{\left(k_{j}+1\right)}-\eta^{\left(k_{j}\right)}∥)=0, \end{array}$$

*and thus, ${\left\{\mu^{\left(m\right)},\phi^{\left(m\right)},\eta^{\left(m\right)}\right\}}_{k=1}^{\infty }$ has a subsequence which converges to the stationary point.*

## 3. Simulation

In this part of the study, simulation experiments were conducted to compare the performance of the extended and classical ADMM clustering algorithms in terms of computational time and clustering accuracy, using the evaluation criterion in (14). The Lasso-based fusion method often leads to the formation of dense clusters with a minor penalty for small differences in $∥\phi_{j}∥$, which can result in the formation of many spurious clusters with very small differences among them [6]. In contrast, the concave penalty method tends to produce a clear cluster structure and a well-defined number of clusters [8]. Therefore, in this study, we focus on the MCP-based fusion method [27] which compares the conventional ADMM’s clustering performance and the proposed new ADMM algorithm.

### 3.1. Low-Dimensional Setting

**Figure 2:**
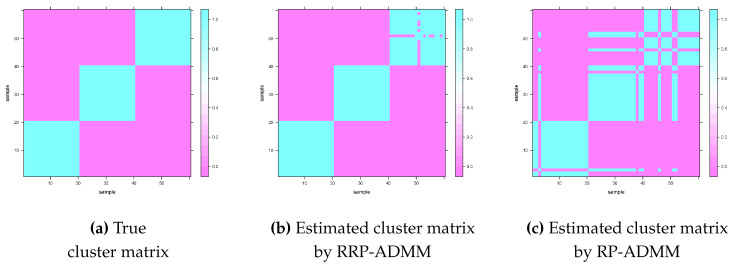
The level plots of cluster matrix including the true one in the left panel, estimators calculated from RRP-ADMM and RP-ADMM in the middle and right panels, respectively.

In this part, we evaluated the clustering performance of the classical ADMM, RP-ADMM, and RRP-ADMM algorithms on low-dimensional synthetic data generated from three overlapping convex clusters with the same spherical shape in some number of dimensions *p* and sample size *n*. The synthetic data were generated from three populations $P_{k}=N\left(\rho_{k},\Sigma \right)$, k=1,⋯,K with K=3, $\rho_{1}=3_{p}$, $\rho_{2}=0_{p}$, $\rho_{3}=-3_{p}$ and $\Sigma ={\left(\sigma_{kj}\right)}_{p\times p}$ with $\sigma_{jj}=1$ and $\sigma_{kj}=0.1^{\left|k-j\right|}$ for k≠j. This setting was chosen deliberately to allow overlap in the sample sets generated from clusters proximal to each other, thereby increasing the complexity of the clustering task. As illustrated in Figure 2c, the clustering performance using a single random projection (RP-ADMM) was suboptimal, indicating challenges with cluster separability under this setup. Conversely, Figure 2b demonstrates that recursive random projection (RRP-ADMM) significantly improved clustering results. The recursive times for the RP-ADMM and RRP-ADMM algorithms were set to M=10.

To evaluate the accuracy of the RP-ADMM, relax-and-split approach [33] (RS-ADMM) and RRP-ADMM algorithms in recovering the true cluster matrix, we generated a random sample of n=60 observations with 1–20 drawn from $P_{1}$, 21–40 drawn from $P_{2}$, and 41–60 drawn from $P_{3}$, and set the number of dimensions to p=5. The probability α of generating a 1 in the random matrix was set to $\alpha =c\frac{\log(n)}{n}$, where *c* controls the probability size. The level plots in Figure 2 use colour to visualize the values of 1’s and 0’s in the cluster matrix. The results show that both RP-ADMM and RRP-ADMM can accurately recover the true cluster matrix, with RRP-ADMM showing more accurate gradation than the true cluster matrix. Single random projection (RP-ADMM) can cause high variance in clustering outcomes due to the randomness of the sampling process. To mitigate this issue, we have adopted the voting-based pooling technique [31], which reduces variance by averaging results from recursive random projection (RRP-ADMM).

**Figure 3:**
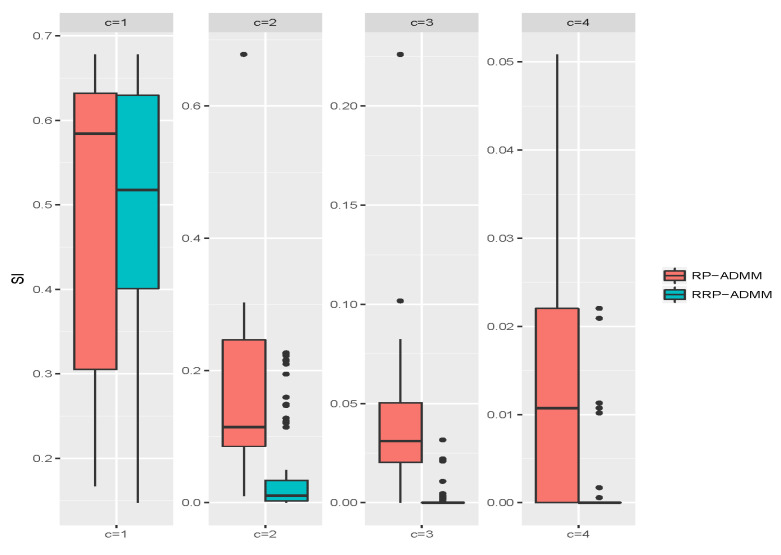
Boxplots of SI values through RP-ADMM and RRP-ADMM algorithms, respectively, under four choices of *c* after 100 replicates.

To further evaluate the performance of the algorithms, we calculated the values of the index SI defined in (14) after 100 replicates under different *c* choices. We depicted the results as boxplots in Figure 3. These results show that RRP-ADMM consistently improves clustering accuracy compared to RP-ADMM, as evidenced by the smaller median and standard error of SI values.

Next, we will compare the performance of classical ADMM and RRP-ADMM in terms of computation time per iteration and the SI after 100 trials. The sample size is varied with n=60,150,240,360 points and $\alpha =4\frac{\log(n)}{n}$, while p=2 is kept constant. In this study, we have limited the number of points to 360, as the classical ADMM algorithm requires a significant amount of computation time for a single realization with more points. We will also compare the performance of the Similarity Index (SI) and Rand Index (RI) in evaluating the clustering results. Therefore, we should calculate the partitioning structure of all points based on the estimated cluster matrix graph. This process involves first identifying the point $a_{1}$ with the most neighbors and aggregating the connected points with point $a_{1}$ as cluster 1, then finding the second point $a_{2}$ with the most edges to form cluster 2, and repeating this process until there are no more points remaining.

**Figure 4:**
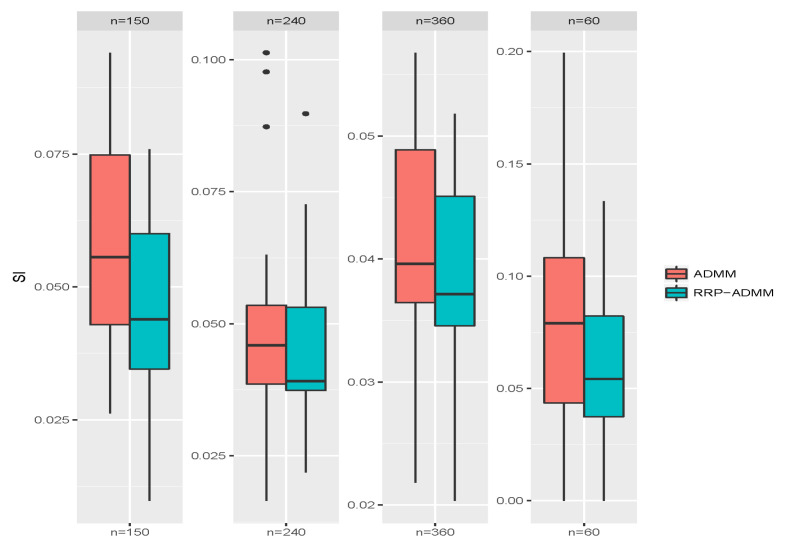
Boxplots of SI values through classical ADMM and RRP-ADMM algorithms, respectively, under four choices of sample sizes *n* after 100 replicates.

**Figure 5:**
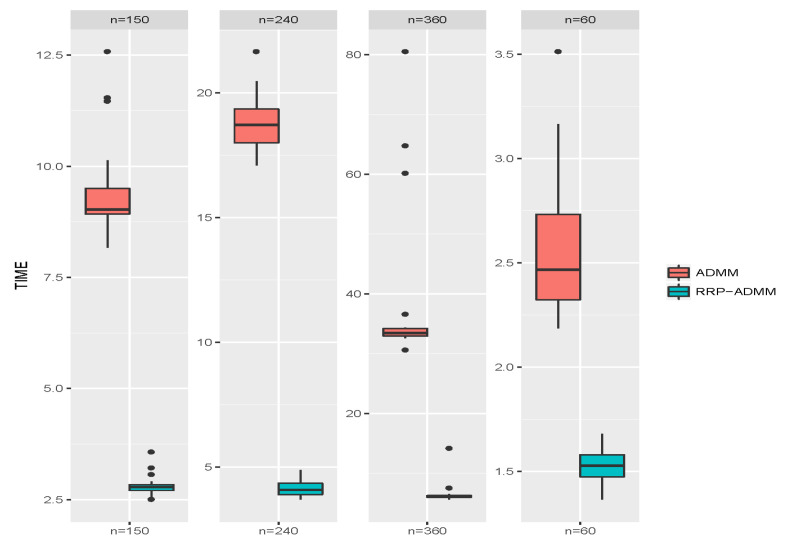
Boxplots of the square root of the run time through classical ADMM and RRP-ADMM algorithms, respectively, under four choices of sample sizes *n* after 100 replicates.

Table 1 shows the mean values of the SI, RI, and the consumed time in seconds for different sample sizes under different methods after 100 replicates. Based on the data in Table 1, we can observe the following: (i) The proposed RRP-ADMM significantly reduces the time required for convex or concave fusion clustering, especially when the sample size increases. (ii) RRP-ADMM produces smaller SI and larger RI values, possibly due to the voting-based pooling technique improving cluster accuracy. (iii) As the sample size increases, the SI and RI values decrease. The boxplots in Figure 4 and Figure 5 demonstrate the superiority of the RRP-ADMM algorithm over the classical ADMM algorithm in terms of both the SI values and the square root of run time, as seen in the results obtained from 100 replicates with four different sample sizes. These results further reinforce our belief in the effectiveness of the RRP-ADMM algorithm.

### 3.2. High-Dimensional Setting

In this part, we investigate using the double random projection-based alternating direction method of multiplier (DRP-ADMM and DRRP-ADMM) algorithms for clustering high-dimensional data sets. We employ a recursive Gaussian distribution-based random projection strategy in the first step to mitigate the impact of randomness on cluster results. Since the classical ADMM algorithm is computationally intensive in high-dimensional settings, we focus on evaluating the performance of the DRP-ADMM and DRRP-ADMM algorithms with recursive times M=9, using three Gaussian random projections in the outer layer and three binary random projections in the inner layer. The simulated data sets consist of two overlapping convex clusters with the same spherical shape. They are generated using a population $P_{k}=N\left(\rho_{k},\Sigma \right)$, k=1,2 with $\rho_{1}=1_{p}$, $\rho_{2}=-1_{p}$. Furthermore, $\Sigma ={\left(\sigma_{kj}\right)}_{p\times p}$ with $\sigma_{jj}=1$ and $\sigma_{kj}=0.1^{\left|k-j\right|}$ for k≠j. We consider four high-dimensional cases with p=1000,2000,3000,5000 and a fixed sample size of n=100.

**Figure 6:**
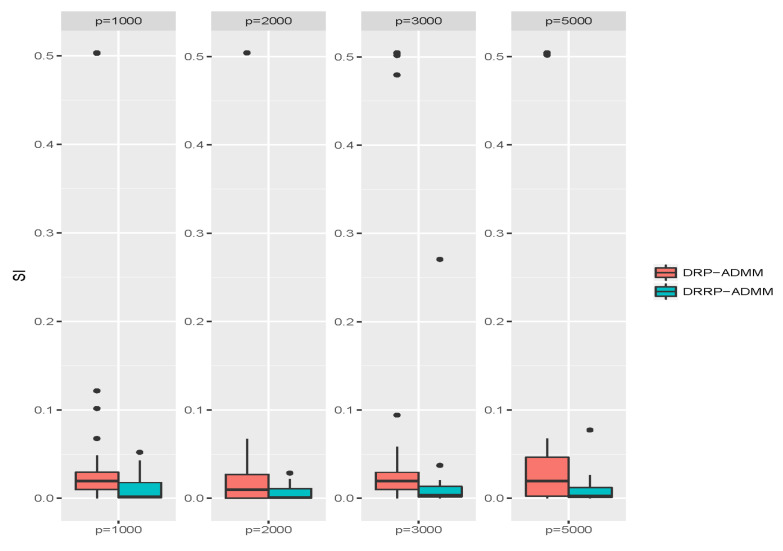
Boxplots of SI values through DRP-ADMM and DRRP-ADMM algorithms, respectively, under four choices of dimensions *p* after 100 replicates.

We evaluate the accuracy of the DRP-ADMM and DRRP-ADMM algorithms in recovering the true cluster matrix. To do this, we first generate a Gaussian random matrix R with dimensions p×q in the first projection. The elements of R correspond to $N(0,1/\sqrt{q})$. We set $q=\lceil \frac{\kappa }{\varepsilon^{2}/2-\varepsilon^{3}/3}\log\left(n\right)\rceil$ with ε=1 and $\kappa =\frac{5}{6}$. See [21,23] for the number of projections. In the second step, we generate a diagonal binary random matrix with probability $\alpha =4\frac{\log(n)}{n}$ of equaling one. Then, we calculate the values of the SI index defined in Equation (14) and plot the results as boxplots in Figure 6 after 100 replicates for different values of *p*. The results show that the DRRP-ADMM algorithm consistently outperforms the DRP-ADMM algorithm regarding the median and standard error of the SI values for all values of *p*, indicating that the DRRP-ADMM algorithm improves clustering accuracy.

## 4. Real Data Analysis

**Figure 7:**
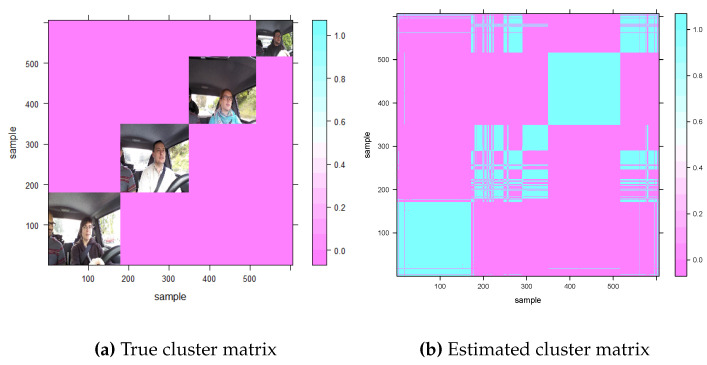
True (**a**) and estimated (**b**) cluster matrix in DrivFace data.

In this study, we use the DrivFace dataset to demonstrate the effectiveness of our proposed clustering procedure. The DrivFace database consists of n=606 images of 640,480 pixels each, captured from four drivers (two women and two men) over different days and containing p=17 facial features such as glasses and beards. Each driver’s images containing similar facial features can be grouped into one cluster, resulting in a total of K=4 clusters as shown in Figure 7a. Firstly, we know the true labels of the dataset; that is, there are four clusters, and we also know which observations belong to the common cluster. Secondly, because the similarity among observations in the pictures is very high across different clusters, it is challenging to separate them. Therefore, we can use this dataset to evaluate our proposed clustering method.

**Figure 8:**
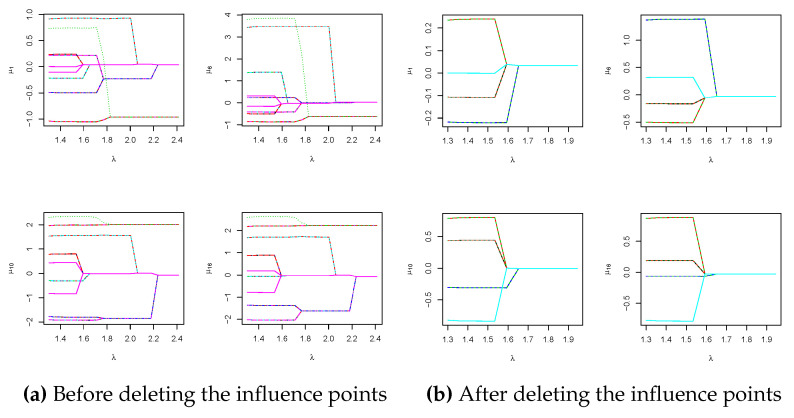
The above fusiongrams are plotted from 4 selected variables in DrivFace data before (left panel) and after (right panel) deleting the influence points, respectively.

Due to the large sample size of the DrivFace dataset, we do not use the classical ADMM algorithm, which would require 606×(606−1)×17/2 operations in a single ADMM iteration. Instead, we first scale the samples by each feature and apply the RP-ADMM procedure to estimate individual centers using a grid of λ values. We plot the fusiongrams of four selected variables in Figure 8, and the scrutiny of Figure 8a implies that some outlying points (influential points) cause the clusters to be dense. We then remove these 55 points and plot a new fusiongram in Figure 8b. The optimal λ value, as determined by the developed BIC criterion in Equation (12), is 1.38, indicating that the estimated number of clusters is four, the same as the number of drivers. We apply the proposed RRP-ADMM algorithm with a Bernoulli-distribution-based random projection procedure to further improve the cluster accuracy using α=10log(n)/n and a recursive number M=20. Using the estimated optimal tuning parameter of 1.38, we obtain the estimated cluster matrix in Figure 7b, which closely resembles the true cluster matrix in Figure 7a. The calculated similarity index (SI) value is 0.098. Moreover, the value of Adjusted Rand Index (ARI) is 0.672.

## 5. Conclusions

We propose using the recursive random projection-based ADMM (RRP-ADMM) method to improve the speed and accuracy of convex and nonconvex fusion clustering. In simulations and real data examples, the RRP-ADMM method demonstrates superior performance in fast calculation and accurate clustering results. The RRP-ADMM algorithm is scalable and can be applied to deal with heterogeneous issues in any setting that involves fusion techniques.

However, some challenges still need to be addressed in this field. One challenge is efficiently transforming the cluster matrix graph into the target partitioning structure and determining the optimal number of clusters. Another challenge is using prior information about which points are more likely to be integrated into a single cluster to reduce the number of pairwise comparisons. Additionally, a further study is needed to determine the theoretical probability of achieving a probability of one in binary random projection. Another future research direction involves performing clustering simultaneously with feature selection, using techniques such as incorporating feature weights [34] or introducing sparsity [14].

## Figures and Tables

**Table 1:** The mean values of Similarity index (SI), Rand Index (RI) and run time in seconds against different sample sizes and different methods after 100 replicates.

	ADMM				RRP-ADMM				RS-ADMM		
**Sample Size**	**SI**	**RI**	**Time**		**SI**	**RI**	**Time**		**SI**	**RI**	**Time**
n=60	0.081	0.921	7		0.059	0.933	2		0.080	0.925	10
n=150	0.058	0.945	88		0.046	0.957	7		0.056	0.947	121
n=240	0.049	0.962	352		0.045	0.974	17		0.047	0.966	551
n=360	0.042	0.973	1582		0.040	0.986	41		0.042	0.978	1864

Note: ‘SI’ represents the similarity index defined in (14), ‘RI’ denotes Rand Index [32]. TIME is the required time in seconds in a single round of ADMM.

## Data Availability

The DrivFace dataset is publicly available at UC Irvine Machine Learning Repository https://archive.ics.uci.edu/dataset/378/drivface, accessed on 11 March 2024.

## Appendix A. Proof of Lemma 1

By the objection function,

(A1) $$\begin{array}{c} Q\left(\mu^{\left(m+1\right)},\phi^{\left(m+1\right)},\eta^{\left(m+1\right)}\right)-Q\left(\mu^{\left(m+1\right)},\phi^{\left(m+1\right)},\eta^{\left(m\right)}\right)=\psi {∥\eta^{\left(m+1\right)}-\eta^{\left(m\right)}∥}^{2} \end{array}$$

and

(A2) $$\begin{array}{c} Q\left(\mu^{\left(m+1\right)},\phi^{\left(m+1\right)},\eta^{\left(m\right)}\right)-Q\left(\mu^{\left(m+1\right)},\phi^{\left(m\right)},\eta^{\left(m\right)}\right)\leq 0 \end{array}$$

Moreover, $\mu \mapsto Q(\mu ,\phi^{\left(m\right)},\eta^{\left(m\right)})$ is strongly convex, as the Hessian matrix ($\psi \Omega^{T}\Omega +I_{np}$) is positive definite, and there exists a constant c>0 such that the following inequality holds:

(A3) $$\begin{array}{c} Q\left(\mu^{\left(m+1\right)},\phi^{\left(m\right)},\eta^{\left(m\right)}\right)-Q\left(\mu^{\left(m\right)},\phi^{\left(m\right)},\eta^{\left(m\right)}\right)\leq -\frac{c}{2}{∥\mu^{\left(m+1\right)}-\mu^{\left(m\right)}∥}^{2} \end{array}$$

Summing (A1)–(A3), we have the result of the above Lemma. In order to prove that the sequence ${\left\{\mu^{\left(m\right)},\phi^{\left(m\right)},\eta^{\left(m\right)}\right\}}_{k=1}^{\infty }$ is convergent, we need to assume that $\phi^{\left(m\right)}$ is bounded and $\psi ∥\eta^{\left(m+1\right)}-\eta^{\left(m\right)}∥\to 0$, which are often observed in numerical tests.

## Appendix B. Proof of Theorem 1

Since ${\left\{\phi^{\left(m\right)}\right\}}_{k=1}^{\infty }$ are bounded, $\mu^{\left(m\right)}$ is also bounded. So $Q(\mu^{\left(m\right)},\phi^{\left(m\right)},\eta^{\left(m\right)})$ and ${\left\{\mu^{\left(m\right)},\phi^{\left(m\right)},\eta^{\left(m\right)}\right\}}_{k=1}^{\infty }$ are bounded. For convenience, we note

$$\begin{array}{cc} \; & L^{\left(m\right)}:=Q\left(\mu^{\left(m\right)},\phi^{\left(m\right)},\eta^{\left(m\right)}\right),\\ \; & y^{\left(m\right)}:=\frac{c}{2}{∥\mu^{\left(m+1\right)}-\mu^{\left(m\right)}∥}^{2},\\ \; & z^{\left(m\right)}:={∥\eta^{\left(m+1\right)}-\eta^{\left(m\right)}∥}^{2}. \end{array}$$

Since $L^{\left(m\right)}$ is bounded, there exist a subsequence $\left\{L^{\left(k_{j}\right)}\right\}$, such that

$$\begin{array}{c} \lim_{k_{j}\to \infty }L^{\left(k_{j}\right)}=\underset{k\to \infty }{lim inf}L^{\left(m\right)} \end{array}$$

By Lemma 1 and $\lim_{k\to \infty }z^{\left(m\right)}\to 0$, we have

$$\begin{array}{cc} \underset{k_{j}\to \infty }{lim inf}y^{\left(k_{j}\right)} & \leq \underset{k_{j}\to \infty }{lim inf}\left(L^{\left(k_{j}\right)}-L^{\left(k_{j}+1\right)}+z^{\left(k_{j}\right)}\right)\\ \; & =\underset{k\to \infty }{lim inf}L^{\left(m\right)}-\underset{k_{j}\to \infty }{lim inf}L^{\left(k_{j}+1\right)}\leq 0. \end{array}$$

As $y^{\left(k_{j}\right)}\geq 0$, ${lim inf}_{k_{j}\to \infty }y^{\left(k_{j}\right)}=0$, which means

$$\begin{array}{c} \underset{k_{j}\to \infty }{lim inf}∥\mu^{\left(k_{j}+1\right)}-\mu^{\left(k_{j}\right)}∥=0, \end{array}$$

together with $∥\eta^{\left(m+1\right)}-\eta^{\left(m\right)}∥\to 0$, we have

$$\begin{array}{c} \underset{k_{j}\to \infty }{lim inf}∥\phi^{\left(k_{j}+1\right)}-\phi^{\left(k_{j}\right)}∥=0. \end{array}$$

The sequence ${\left\{\mu^{\left(m\right)},\phi^{\left(m\right)},\eta^{\left(m\right)}\right\}}_{k=1}^{\infty }$ have a subsequence ${\left\{\mu^{\left(k_{j}\right)},\phi^{\left(k_{j}\right)},\eta^{\left(k_{j}\right)}\right\}}_{k_{j}=1}^{\infty }$ which converges to a point $\left\{\mu^{*},\phi^{*},\eta^{*}\right\}$. Then, we have

$$\Omega_{j}\mu^{*}-\phi_{j}^{*}=0,1\leq j\leq \kappa .$$

Moreover, the procedure to solve the objective function satisfies the following optimality system:

$$\begin{array}{c} \left\{\begin{array}{c} \mu^{\left(m+1\right)}-X+\psi \Omega^{T}\left(\Omega \mu^{\left(m+1\right)}-\phi^{\left(m\right)}+\frac{\eta^{\left(m\right)}}{\psi }\right)=0,\\ 0\in -\psi \left(\Omega_{j}\mu^{\left(m+1\right)}-\phi_{j}^{\left(m+1\right)}+\frac{\eta_{j}^{\left(m+1\right)}}{\psi }\right)+\frac{\partial p_{\lambda }(∥\phi_{j}∥)}{\partial \phi_{j}}{|}_{\phi_{j}=\phi_{j}^{\left(m+1\right)}}.\; \end{array}\right. \end{array}$$

So,

$$\begin{array}{c} \left\{\begin{array}{c} \mu^{*}-X-\Omega^{T}\eta^{*}=0,\\ 0\in -\eta_{j}^{*}+\frac{\partial P_{\lambda }(∥\phi_{j}∥)}{\partial \phi_{j}}{|}_{\phi_{j}=\phi_{j}^{*}}. \end{array}\right. \end{array}$$

Therefore, $\left\{\mu^{*},\phi^{*},\eta^{*}\right\}$ is a KKT point of objective function. We complete the proof.
